# Vertebrate scavenger guild composition and utilization of carrion in an East Asian temperate forest

**DOI:** 10.1002/ece3.5976

**Published:** 2020-01-21

**Authors:** Akino Inagaki, Maximilian L. Allen, Tetsuya Maruyama, Koji Yamazaki, Kahoko Tochigi, Tomoko Naganuma, Shinsuke Koike

**Affiliations:** ^1^ Graduate School of Agriculture Tokyo University of Agriculture and Technology Fuchu Tokyo Japan; ^2^ Illinois Natural History Survey University of Illinois Champaign IL USA; ^3^ Nature Preservation Division Tochigi Prefecture Utsunomiya Tochigi Japan; ^4^ Department of Forest Science Tokyo University of Agriculture Setagaya Tokyo Japan; ^5^ United Graduate School of Agricultural Science Tokyo University of Agriculture and Technology Fuchu Japan; ^6^ Institute of Agriculture Tokyo University of Agriculture and Technology Fuchu Japan; ^7^ Institute of Global Innovation Research Tokyo University of Agriculture and Technology Fuchu Japan

**Keywords:** carcass, *Cervus nippon*, facultative scavenging, feeding behavior, scavengers

## Abstract

Scavenging is a common feeding behavior by many species that plays an important role in ecosystem stability and function while also providing ecosystem services. Despite its importance, facultative scavenging on large animal carcasses has generally been overlooked in Asian temperate forest ecosystems. The aim of this study was to determine the composition and feeding behavior of the facultative scavenger guild as it relates to sika deer (*Cervus nippon*) carcasses in Japanese forests. There are no obligate scavengers or large predators that kill adult ungulates, but humans fill the role of large predators by culling deer for population management. We documented nine vertebrate species scavenging on deer carcasses and found that mammals were more frequent scavengers than birds and also fed for longer durations. This result suggests that there is a facultative scavenger guild composed mainly of mammals in our forest ecosystem and that carcass utilization by birds was restricted to only forest species. Raccoon dogs (*Nyctereutes procyonoides*) and Asian black bears (*Ursus thibetanus*) were the most frequent scavenger species and also fed for longer durations than other scavengers. There were significant seasonal differences in scavenging by Asian black bear, Japanese marten (*Martes melampus*), and mountain hawk‐eagle (*Nisaetus nipalensis*), suggesting the availability of other food resources may alter facultative scavenging by each species. Our results support that scavenging is widespread in this system and likely has important functions including building links in the food web.

## Graphical abstract

This study is the first to document the composition of the vertebrate scavenger guild and how scavengers utilize deer carcasses in Asian temperate forest ecosystems. We found the facultative scavenger guild was primarily composed of omnivorous mammals, and raccoon dog (*Nyctereutes procyonoides*) and Asian black bear (*Ursus thibetanus*) were the most frequent scavenger species. Our results support that scavenging is widespread in this system and likely has important functions including building links in the food web.

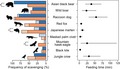

## 1 INTRODUCTION

Scavenging is a common feeding behavior in which scavengers compete for ephemeral but nutritious resources in the form of vertebrates carcasses (Sebastián‐González et al., 2019; Wilson & Wolkovich, 2011). Various biological groups from decomposers (e.g., invertebrates and microbes) to vertebrate scavengers compose the characteristic communities that interact at carcasses (DeVault, Rhodes, & Shivik, 2003). The vertebrate scavenger guild includes obligate scavengers (i.e., vultures) that are dependent on carrion and facultative scavengers (primarily carnivores and raptors) that opportunistically feed on carrion (Beasley, Olson, & DeVault, 2015). Vertebrate scavenging plays an important role in ecosystem stability by cycling nutrients and shaping food web links that originate from carcasses (Selva & Fortuna, 2007; Wilson & Wolkovich, 2011). Scavengers also provide important ecosystem services by removing carcasses that become reservoirs for potentially harmful bacteria and pathogens from ecosystems (DeVault et al., 2003; Inger, Cox, Per, Norton, & Gaston, 2016; Moleon et al., 2014).

Vertebrate facultative scavengers build several “weak links” within food webs by feeding on different species (DeVault et al., 2003; Wilson & Wolkovich, 2011), and these weak links are essential for ecosystem stability and maintenance (McCann, Hastings, & Huxel, 1998; Neutel, Heesterbeek, & Ruiter, 2002). The majority of scavengers in most ecosystems are facultative rather than obligate (Wilson & Wolkovich, 2011), making it important to evaluate the complex interactions between facultative scavengers at carrion to understand their function in ecosystem dynamics (Cortés‐Avizanda, Selva, Carrete, & Donázar, 2009; Sebastián‐González et al., 2016; Selva & Fortuna, 2007). Nevertheless, few studies have dealt with facultative scavenging activities in the Asian temperate forest ecosystem (e.g., Sugiura, Tanaka, Taki, & Kanzaki, 2013, Sugiura & Hayashi, 2018) and there have been no studies focused on facultative scavenger guilds at large ungulate carcasses.

Japan (East Asian islands, approximately 378,000 km^2^) is designated as an ecosystem hot spot with high biodiversity (Mittermeier, Myers, Mittermeier, & Robles, 1999) and has stable forest ecosystems that provide habitat for various, densely populated species of mammals. On the other hand, the forest ecosystem contains overpopulated and increasing large ungulate (sika deer, *Cervus nippon*, hereafter “deer”; and wild boar, *Sus scrofa*) populations that need to be managed by humans. Mainland Japan, however, contains no large predators that will kill adult ungulates (Asian black bear *Ursus thibetanus* is the only large carnivore found in mainland Japan, but does not normally kill adult ungulates and has an omnivorous feeding strategy) or any obligate scavengers. However, humans fill the role of large predators by culling of ungulates for population control throughout the year. For example, approximately 850,000 ungulates (440,000 deer and 410,000 wild boar) were culled throughout the year and approximately 304,000 ungulates were hunted in the recreational hunting season (basically from 15 November to 15 February) in 2017 (Ministry of the Environment, 2019). Wild game are not commonly consumed in Japan, and the meat of ungulates culled during the nonrecreational hunting season (i.e., spring to autumn) are typically discarded. The ungulates culled in mountain areas are often left or buried in the forest because it is difficult to move them out of the forest. The other cause of ungulate mortality is starvation in late winter and early spring (Takatsuki, Suzuki, & Suzuki, 1994), but there have been no studies that quantified the natural death of ungulates in Japan.

Carrion from various sources, including natural mortality, kills by large carnivores, and carcasses left behind by humans, are known to be an important and readily used food resource for facultative scavengers (Pereira, Owen‐Smith, & Moleón, 2014; Selva, Jędrzejewska, Jędrzejewski, & Wajrak, 2005). Carrion from large carnivores is readily exploited by the scavenger guild (Allen, Elbroch, Wilmers, & Wittmer, 2015; Selva & Fortuna, 2007; Wilmers, Crabtree, Smith, Murphy, & Getz, 2003) and is often easier to find than carrion from natural mortality (Selva & Fortuna, 2007). Carrion from carcasses left behind by humans, either remains from hunters or entire culled animals, is also readily used (Gomo, Mattisson, Hagen, Moa, & Willebrand, 2017; Selva & Fortuna, 2007; Wilmers, Stahler, Crabtree, Smith, & Getz, 2003). Scavenger guild assemblages and richness vary among habitats (e.g., Sebastián‐González et al., 2019), but appear to vary less when considering the source of carrion (e.g., Selva & Fortuna, 2007). The availability of ungulate carcasses from culling in Japan should therefore be an important resource for the facultative scavenger guild, and monitoring these carcasses may be an informative method of determining if scavengers consume large ungulate carrion in Japan.

The objective of this study was to determine the composition of the vertebrate scavenger guild and how scavengers utilize Sika deer carcasses in the Asian temperate forest. We used video camera traps to evaluate the following specific questions and hypotheses: (a) What is the composition of vertebrate species that scavenged from deer carcasses? We hypothesized that omnivorous mammals and birds in the ecosystem would scavenge opportunistically. However, we also hypothesized that mammals and birds would have different frequencies of scavenging and feeding durations because of differences in morphology, their ecology, and energetic needs. (b) How do scavenger species vary in their frequency of carcass use? We hypothesized that raccoon dogs (*Nyctereutes procyonoides*) and masked palm civets (*Paguma larvata*) would be the most frequent scavengers based on previous research in Japan (Sugiura et al., 2013). (c) How do scavenger species vary in their feeding durations on the carcasses? We hypothesized that Asian black bears would feed for longer durations than other scavengers based on bears being dominant scavengers in other ecosystems (Allen et al., 2015; Krofel, Kos, & Jerina, 2012). We also hypothesized that raccoon dogs might scavenger for longer durations than other scavengers due to being the most frequent scavenger in other studies in Japan (Sugiura & Hayashi, 2018; Sugiura et al., 2013) and therefore possibly more dependent on carrion than other species. (d) Does the frequency or duration of scavenger species vary among the two seasons—summer and autumn? We hypothesized that some species would change their utilization of the carcasses seasonally based on energetic needs and the changing availability of their primary foods because most potential vertebrate scavengers are omnivores.

## 2 MATERIALS AND METHODS

### 2.1 Study area

Our research was conducted in Nikko National Park, central Japan (approximately 1,150 km^2^; 36°36′N–37°05′N, 139°19′E–139°51′E; elevation: 300–1,300 m). At the Okunikko metrological station, in the center of national park, the mean annual temperature was 7.7°C (−12.9 to 27.7°C) and the mean annual rainfall was 163.1 mm (Japan Meteorological Agency, 2016, 2017). The forest types included deciduous broadleaved forests (comprised mainly of *Quercus serrata*, *Q. crispula Blume*, and *Cerasus jamasakura*), conifer plantation forests (comprised mainly *Cryptomeria japonica, Chamaecyparis obtuse*, and *Larix kaempferi*), and also patchy mixed forests. The forest floor consisted primarily of bamboo grasses in each forest type.

The most conspicuous terrestrial mammals that feed on animal matter and are expected to scavenge are the Asian black bear, wild boar, red fox (*Vulpes vulpes*), raccoon dog, Japanese badger (*Meles anakuma*), Japanese weasel (*Mustela itatsi*), masked palm civet, and Japanese marten (*Martes melampus*) (Ohdachi, Ishibashi, Iwasa, & Saitoh, 2009; Sugiura et al., 2013; Tochigi Prefecture, 2002). None of these mammals are predators of large, adult ungulates, although the Asian black bear is known to kill neonatal deer (Hashimoto & Takatsuki, 1997). Bird species that are known to feed on vertebrates in the forest and may be expected to scavenge are the bull‐headed shrike (*Lanius Bucephalus*), Japanese sparrowhawk (*Accipiter gularis*), Sparrowhawk (*Accipiter nisus*), goshawk (*Accipiter gentilis*), jungle crow (*Corvus macrorhynchos*), carrion crow (*Corvus corone*), black kite (*Milvus migrans*), Japanese scops owl (*Otus scops*), Ural owl (*Strix uralensis*), Gray‐faced buzzard (*Butastur indicus*), Eastern Buzzard (*Buteo japonicus*), Golden eagle (*Aquila chrysaetos*), and mountain hawk‐eagle (*Nisaetus nipalensis*) (Higuchi, Morioka, & Yamagishi, 1996, 1997; Maki & Ohnishi, 2001; Tochigi Prefecture, 2001; Wild Bird Society of Japan‐Tochigi, 2015).

### 2.2 Data collection

We obtained 42 fresh deer carcasses, that had not previously been scavenged, from culled nuisance animals or animals killed through vehicle collisions from June to November in 2016 (*n* = 20 deer) and 2017 (*n* = 22 deer). Our handling of the carcasses was conducted in accordance with the guidelines for use and collection of animals in research established by the Mammal Society of Japan (2009). We used the culling method that most minimizes pain and distress to the animal, in accordance with the “Welfare and Management of Animals Act” (Ministry of the Environment). These culls were conducted for the study with permission from the Tochigi Prefecture Government according to “Wildlife Protection and Proper Hunting Act” (Ministry of the Environment) and “Specified Wildlife Conservation and Management Plan” (Tochigi Prefecture, 2018), both of which cover the controlled culling of deer. We used deer of various sizes, from newborn to adult.

We placed each deer carcass on the ground near the culled location in mature deciduous broadleaved forests and patchy mixed forests. Each carcass was placed at a distance of >1 km from other carcasses when we obtained the deer carcasses during the same period. In some cases (*n* = 6), we placed carcasses within a radius of 500 m of previous carcasses from 1 to 4 weeks of the previous carcass. In these cases, the previous deer carcass was consumed in 5.1 d (±1.9 SE, range 3.4–9.0 d), whereas at the other 42 carcasses the carcasses persisted for 6.8 d (±4.0 SE, range 2.3–16.5 d). In addition, the time between placing subsequent deer carcasses within a radius of 500 m was >1 week. The locations were on private lands within the park where landowners had given us permission to place the deer carcasses. We secured the deer carcasses to the nearest tree using wire rope to prevent them from being removed from the view of the camera trap by scavengers.

We monitored the carcasses using Ltl Acorn 6210 camera traps (Green Bay, Wisconsin, USA). We set the camera traps 2–5 m from the deer carcass and 1–2 m above ground. We programmed the camera traps to record 30‐s videos at each trigger with a 30‐s refractory period. We stopped monitoring a carcass when >80% of the deer carcass including bones and skins had been consumed, because there was little available carrion or nutrition for facultative vertebrate scavengers remaining.

### 2.3 Statistical analyses

To understand the scavenger guild and utilization, we identified each vertebrate scavenger species that we documented scavenging from carcasses and classified vertebrate scavengers as either mammalian or avian classes. We excluded any species that passed through the view of the camera trap inadvertently. We used program R 3.2.4 (R Core Team, 2016) for all of our statistical analyses.

To evaluate the scavenger guild, we calculated the number of deer carcasses that each scavenger species visited and the number of deer carcasses that each scavenger species scavenged. We used Fisher's exact tests (Fisher, 1934) to determine (a) differences in the percentages of visited carcasses between mammalian and avian scavengers and (b) differences in the percentages of scavenged carcasses between mammalian and avian scavengers.

To evaluate differences in the frequency of visitation and scavenging among scavenger species, we used pairwise comparisons with Fisher's exact tests (Fisher, 1934) using RVAideMemoire package (Hervē, 2019). We determined (a) differences in the percentages of visited carcasses between scavenger species and (b) differences in the percentages of scavenged carcasses between scavenger species.

To evaluate how long scavengers fed on the carcasses, we calculated total feeding times (min) at each deer carcass for each vertebrate scavenger from the video recordings. In cases where there were more than five avian scavengers in one video, it was not possible for us to determine exact feeding times. Instead, we estimated the feeding time of each avian scavenger species in these videos by summing the feeding time of five randomly selected individuals and multiplying this value by the maximum number of individuals during the visit divided by five. In our analyses of feeding times, we first tested for differences in the mean feeding times between the classes of mammalian and avian scavengers using a Wilcoxon rank‐sum test (Sokal & Rohlf, 2012). We then tested for differences in the feeding durations among scavenger species to see if some species scavenged for longer durations than others. We modeled the mean feeding time of all species using a generalized linear model (GLM) with a Gamma distribution. We added 10^–4^ to all of the zero values of the total feeding times (i.e., instances where a species visited a carcass but did not scavenge) to ensure a fit of the Gamma distribution. We used the total feeding times at carcasses as our dependent variable and used the scavenger species as the independent variables. We performed post hoc pairwise comparisons with Tukey's HSD test (Sokal & Rohlf, 2012) using the multcomp package (Hothorn, Bretz, & Westfall, 2008) between each species pair (i.e., 36 comparisons) to determine where significant differences occurred.

To understand differences in scavenging among seasons, we classified the seasons as summer (June to August) and autumn (September to November). We used Fisher's exact test (Fisher, 1934) to examine (a) differences in the percentages of visited carcasses seasonally between mammalian and avian scavengers, (b) differences in the percentages of scavenged carcasses seasonally between mammalian and avian scavengers, and (c) the seasonal differences in the percentages of scavenged carcasses for each species. We then used Wilcoxon rank‐sum test (Sokal & Rohlf, 2012) to examine differences in feeding duration seasonally between mammalian and avian scavengers.

## 3 RESULTS

### 3.1 Scavenger guild

**Figure 1:**
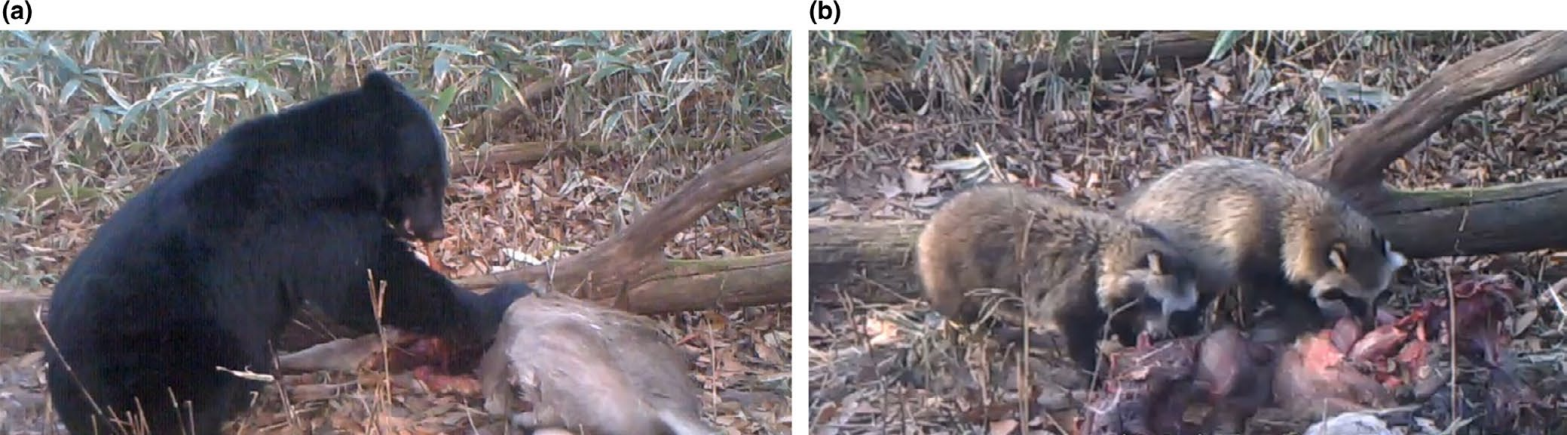
Images (from video recordings) of our most frequent scavenger species (a) Asian black bear and (b) raccoon dogs scavenging on the same sika deer carcass in 8 November 2016

**Figure 2:**
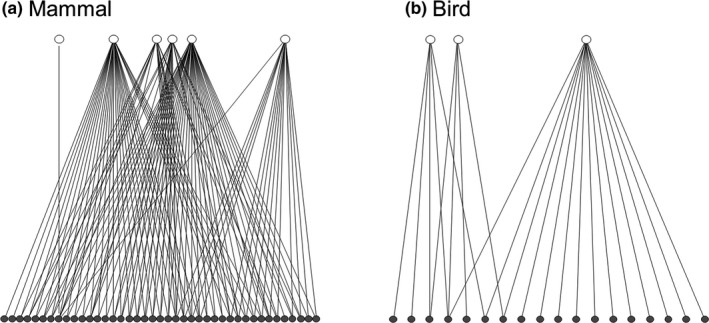
Bipartite graphs depicting scavenging interactions between the species of facultative scavengers (open circles) and deer carcasses (filled circles) of: (a) mammalian and (b) avian scavenger classes. Each line shows a direct scavenging link, where a given scavenger species fed on a particular carcass

We recorded that one or more vertebrate scavengers visited and scavenged every deer carcass in this study. We identified nine facultative scavengers, including six mammalian species (Asian black bear; Figure 1a, wild boar, raccoon dog; Figure 1b, red fox, Japanese marten, and masked palm civet) and three avian species (mountain hawk‐eagle, black kite, and jungle crow). We also documented visitation and scavenging by mammals at a significantly higher number of carcasses than birds (both *p* < .001; Figure 2).

### 3.2 Frequency of scavenging

All scavengers except Asian black bears and wild boars occasionally visited carcasses but did not scavenge (e.g., they exhibited behaviors such as smelling the carcass or exploring the area around the carcass, but did not feed). This was most common in red foxes, Japanese martens, masked palm civets, and jungle crows (Table 1).

**Table 1:** The utilization of sika deer carcasses by vertebrate scavenger species. We report the percentage of carcasses visited and percentage scavenged from overall and in each season, as well as the mean feeding time for each species

Common name	Species	Percentage of visited carcasses (%)^a^			Percentage of scavenged carcasses (%)^a^			Mean of feeding time ± *SD* (m)
		Total (42)	Summer (20)	Autumn (22)	Total (42)	Summer (20)	Autumn (22)	Total (42)
Mammal		100.0	100.0	100.0	97.6	100.0	95.5	80.2 ± 90.0
Asian black bear	*Ursus thibetanus*	73.8	95.0	54.5	73.8	95.0	54.5	36.9 ± 34.9
Wild boar	*Sus scrofa*	42.9	40.0	45.5	42.9	40.0	45.5	21.0 ± 25.4
Raccoon dog	*Nyctereutes procyonoides*	90.5	85.0	95.5	85.7	80.0	90.9	52.6 ± 78.1
Red fox	*Vulpes vulpes*	47.6	30.0	63.6	35.7	20.0	50.0	4.0 ± 8.6
Japanese marten	*Martes melampus*	54.8	35.0	72.7	38.1	20.0	54.5	7.8 ± 12.5
Masked palm civet	*Paguma larvata*	16.7	20.0	13.6	2.4	0.0	4.5	0.9 ± 0.9
Avian		52.4	40.0	63.6	42.9	40.0	45.5	29.3 ± 80.0
Mountain hawk‐eagle	*Nisaetus nipalensis*	14.3	5.0	22.7	11.9	0.0	22.7	23.0 ± 14.4
Black kite	*Milvus migrans*	11.9	10.0	13.6	9.5	10.0	9.1	0.5 ± 0.4
Jungle crow	*Corvus macrorhynchos*	45.2	35.0	54.5	33.3	35.0	31.8	29.0 ± 78.3

a Numbers in parentheses indicate the number of carcasses.

**Figure 3:**
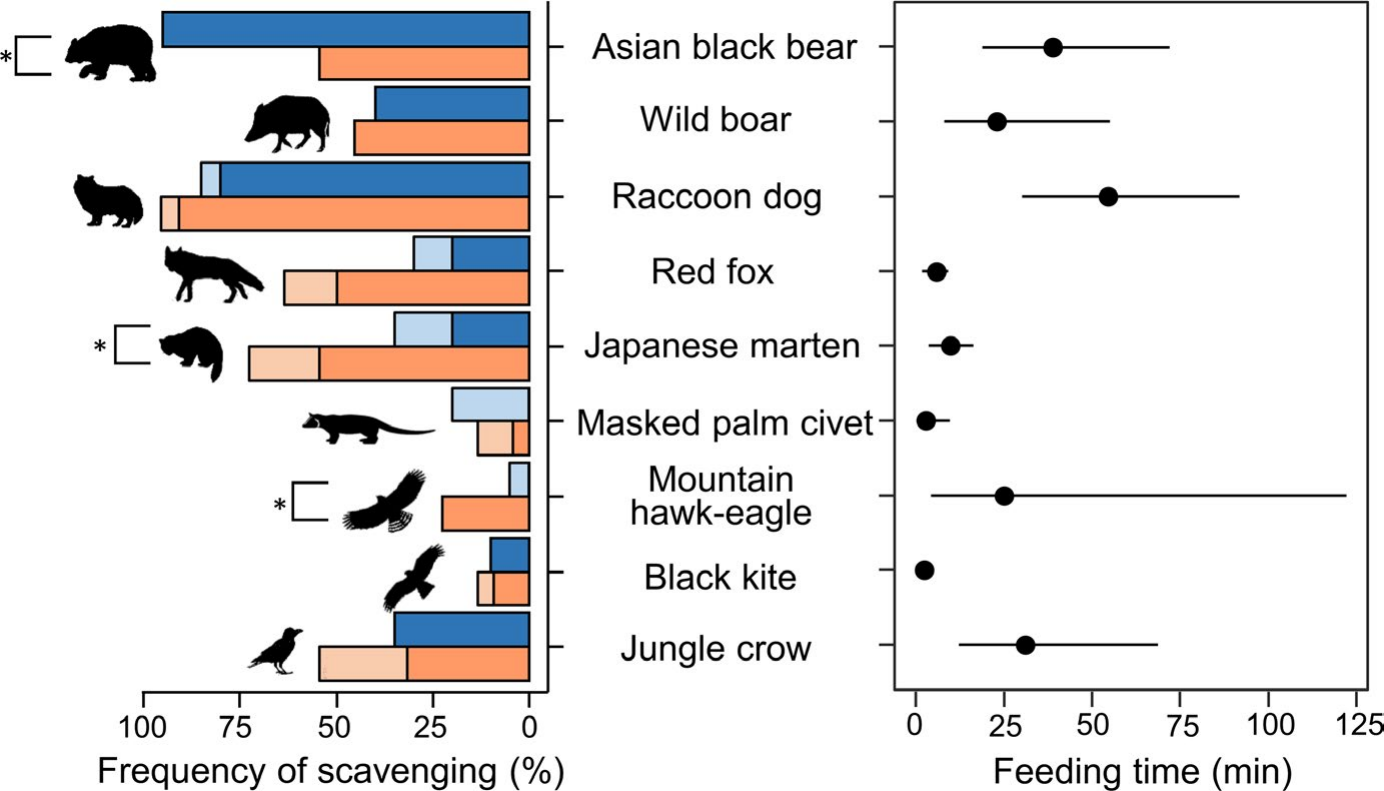
The utilization of deer carcasses for each species. The frequency (%) of scavenging is shown on the left. Blue colors indicate summer, orange colors indicate autumn, light colors indicate visited carcasses, and dark colors indicate scavenged carcasses. The asterisks indicate significant differences between season on the percentage of scavenged carcasses (*p* < .05). Mean feeding time (min) with 95% confidence intervals shown on the right

The most frequent scavenger species were raccoon dogs (85.7%) and Asian black bears (73.8%, Figure 3), which scavenged at a significantly higher number of carcasses than other scavengers (*p* < .001). In contrast, our least frequent scavengers were masked palm civets (2.4%; only scavenged from one carcass), black kites (9.5%), and mountain hawk‐eagles (11.9%, Figure 3). Scavenging by these species was at a significantly lower number of carcasses than other scavengers (*p* < .001). There were no differences in scavenging frequency among wild boars, red foxes, Japanese martens, and jungle crows (*p* > .05). We also found similar results in visitation frequency, but there was not a significant difference between Asian black bears and Japanese martens (*p* = .147).

### 3.3 Feeding durations

The mean feeding time at carcasses was longer for mammals (80.2 ± 90.0 *SD* min) than for birds (29.3 ± 80.0 min; *p* < .001; Table 1).

Among scavenger species, Asian black bears (mean = 36.9 ± 34.9 min) and raccoon dogs (mean = 52.6 ± 78.1 min) fed for the longest durations (Table 1, Figure 1). Asian black bears fed significantly longer than red foxes (β = 2.224; *p* < .01), Japanese martens (β = 1.556; *p* < .05), and black kites (β = 4.268; *p* < .01). Raccoon dogs also fed significantly longer than red foxes (β = 2.579; *p* < .001), Japanese martens (β = 1.911; *p* < .01), masked palm civets (β = 4.068; *p* < .05), and black kites (β = 4.623; *p* < .001). Jungle crows fed significantly longer than red foxes (β = 1.983; *p* < .05), and black kites (β = 4.027; *p* < .01); and wild boars fed significantly longer than black kites (β = 3.704; *p* < .05).

### 3.4 Seasonality of scavenging

We documented visitation and scavenging by mammals at a significantly higher number of carcasses than birds overall in both seasons (*p*_summer_ < 0.001, *p*_autumn_ < 0.001). We also documented mammals also fed for longer durations than bird overall in both seasons (*p*_summer_ = 0.023, *p*_autumn_ < 0.001).

The percentage of scavenged carcasses by Asian black bears decreased significantly from summer to autumn (*p* = .0042), but the percentage of scavenged carcasses by Japanese martens and mountain hawk‐eagles significantly increased (*p*_martens_ = 0.0289, *p*_hawk‐eagles_ = 0.0492, Table 1). Red foxes also increased the scavenging frequency slightly from summer to autumn (*p* = .0577, Table 1). There were no significant differences in frequency of scavenging by season for other scavengers (Table 1).

## 4 DISCUSSION

### 4.1 Characteristics of scavenger guilds

The composition of scavenger guilds vary based on latitude, landscape composition (e.g., habitat), topography, and human footprint (Pardo‐Barquín, Mateo‐Tomás, & Olea, 2019; Sebastián‐González et al., 2019; Turner, Abernethy, Conner, Rhodes, & Beasley, 2017). We documented the facultative scavenger guild in a forest ecosystem in mainland Japan that was composed mainly of omnivorous carnivores, as well as a few forest bird species. Raccoon dogs and Asian black bears are important scavengers in this system, as they fed on more carcasses and for longer durations than other scavengers. Despite the lack of previous studies, we found that scavenging is widespread and likely has important functions in the system (e.g., DeVault et al., 2003; Inger et al., 2016; Moleon et al., 2014), including forming weak links in food webs (e.g., Selva & Fortuna, 2007; Wilson & Wolkovich, 2011).

We recorded only three avian scavengers, mountain hawk‐eagle, black kite, and jungle crow, and we did not detect any nonforest bird species. Worldwide, the composition of the scavenger guild that consumes remains from human hunters is generally dominated by birds (Mateo‐Tomás et al., 2015), but this pattern was the opposite in our study area. These results suggest that carcass utilization and feeding by avian scavengers were restricted because we placed the carcasses in a mature forest. Avian scavengers often use visual cues to detect carcasses (Selva et al., 2005), and in dense forests mammals often consume most carcasses because visual detection of the carcasses by avian scavengers is limited (DeVault & Rhodes, 2002). Thus, the difference in habitat type (e.g., open or closed habitat, various types of forest) directly affects the composition of the scavenger guild (Devault, Seamans, Linnell, Sparks, & Beasley, 2017; Olson, Beasley, & Rhodes, 2016; Pardo‐Barquín et al., 2019; Selva et al., 2005; Turner et al., 2017), and it is important to determine differences in the composition of scavengers among local guilds. In addition, it is likely that the absence of large predators reduces the ability of avian scavengers to detect carcasses in Japan, as avian scavengers efficiently detect and utilize carcasses by tracking large carnivores (Wilmers, Stahler, et al., 2003).

All mammalian scavengers, except the wild boar, were carnivores; however, we did not document either Japanese badgers or Japanese weasels scavenging. We expected Japanese badgers to scavenge because deer hair had previously been found within its scat (Yamamoto, 1994). The Eurasian badger (*Meles meles*) is closely related to the Japanese badger and is known as an infrequent scavenger in European temperate forests (e.g., Ray, Seibold, & Heurich, 2014; Selva et al., 2005; Young, Márquez‐Grant, Stillman, Smith, & Korstjens, 2015). However, the Eurasian badger does not scavenge when its main food resources, such as earthworms and insects, are plentiful (Young et al., 2015), and the Japanese badger also feeds primarily on earthworms and insects (Kaneko, Maruyama, & Macdonald, 2006). Furthermore, in areas of high deer density, such as our study area, the abundance of Japanese badgers often increases with the increase in their primary food sources (Seki, Okuda, & Koganezawa, 2014). This suggests that it may not have been necessary for the Japanese badger to scavenge because of the abundance of invertebrates. We also expected Japanese weasels to scavenge because of previous documentation of scavenging on mouse carcasses (Sugiura & Hayashi, 2018; Sugiura et al., 2013). However, previous diet analysis showed they feed on small vertebrates and not vertebrates that are larger than themselves (e.g., Asahi, 1975; Furuya, Kishida, Swnoo, Noguchi, & Yamasaki, 1979). Thus, Japanese weasels may not consider deer carcasses as food resources. However, Japanese weasels are smaller than other carnivores and are more difficult to document with camera traps, and we may have just failed in detecting their scavenging behavior.

We also have to consider how our study design may have affected our documentation of the facultative scavenger guild. First, we set the deer carcasses at a distance of >1 km from other carcasses when we obtained them around the same time. However, 1 km is a small distance to move between carcasses for some scavengers, especially those with large home ranges. This may affect the frequency and duration of scavenging by individual animals. Second, the time between placing subsequent deer carcasses at the same location was a minimum of 1 week. This relatively short time span risks individual animals learning where the carcasses exist and leads to repeated exploitation (i.e., vulture restaurant; Cortés‐Avizanda, Jovani, Carrete, & Donázar, 2012). Future research is needed to determine how study design affects documentation of scavenger guilds, as established methodologies are often variable for carcass monitoring (distance and time span when the carcass setting, carcass size, how to monitor).

### 4.2 Scavenger frequency and duration

The frequency and duration of scavenging varied among species in our study, potentially due to their ecology or perceived risk from competition. Raccoon dogs and Asian black bears were the most frequent scavengers in our study, having visited and scavenged most of the carcasses and fed for the longest durations. This supports that raccoon dogs are the most frequent scavengers in Japan (Sugiura et al., 2013). We have also shown that Asian black bears have an important role as dominant scavengers (e.g., Allen et al., 2015; Krofel et al., 2012) as the only large carnivores in Japan and may have a greater ability to detect and consume large ungulate carcasses. In contrast, masked palm civets were the least frequent scavenger, only scavenging from one carcass, even though previous studies documented high scavenging frequency (Sugiura et al., 2013). This suggests that they may not scavenge as frequently on large carcasses, which could not be as easily and quickly eaten as small carcasses. This suggests that studies need to account for the effect of scavenging by carcass size or type (Turner et al., 2017). Mountain hawk‐eagle and black kites, who have solitary feeding strategies, were also the least frequent scavengers. This could imply the carcass detection by solitary birds in forest habitat is more difficult than by group birds such as jungle crows.

In many Japanese forest ecosystems, humans directly provide a large amount of carrion through mass culling of ungulates for management. This rapid and pulsed provision of carrion may outweigh previous supplies by large predators (e.g., Japanese wolf *Canis lupus hodophilax* who became extinct in the early 20th century). Such artificial pulses of carrion biomass affect the scavenging patterns of obligate scavengers as well as facultative scavengers in many countries (e.g., Blazquez, Sanchez‐Zapata, Botella, Carrete, & Eguía, 2009; Mateo‐Tomás & Olea, 2010; Mateo‐Tomás et al., 2015; Wilmers, Stahler, et al., 2003). Similarly, the provisioning of the artificial carrion in our system may affect not only facultative scavenging directly but also potential feeding strategies of vertebrate scavengers and activities of various organisms (e.g., invertebrates and microbes) through indirect biological interactions. The culled ungulate carcasses are exploited by vertebrate scavengers and decomposers, but because all scavengers are facultative in the system they may become satiated on ungulate meat during large pulses. Future studies on the effect of carrion from human harvests on ecosystem functions and facultative scavengers are necessary when culling is extensively used for management.

There may be some perceived risk from competition with other scavengers (e.g., Allen, Wilmers, Elbroch, Golla, & Wittmer, 2016; Hunter, Durant, & Caro, 2007; Pereira et al., 2014). For example, some species (especially red foxes, Japanese martens, and jungle crows) visited the carcasses but often did not scavenge from them, and red foxes and martens fed less than bears and raccoon dogs. Previous studies have shown that the size of an animal predicts its ability to compete at carcasses—with larger animals being more competitive (Moleón, Sánchez‐Zapata, Selva, Donázar, & Owen‐Smith, 2014; Pereira et al., 2014). We documented that bears, the largest scavenger, remained for long periods of time near the carcasses and also rubbed on the carcasses repeatedly (Video S1). Meanwhile, mountain hawk‐eagles were never recorded with other species although black kites were recorded with jungle crows on three occasions. These documentations of apparent dominance behaviors may deter other scavengers from scavenging, making competitive interactions among species in our scavenger guild important. However, the competitive interactions could be affected not only by size but also by other factors (e.g., hunger or species‐specific traits; Allen et al., 2016). Further study is necessary to understand these mechanisms that affect the complex competitive interactions in the scavenger guild.

### 4.3 Seasonal differences

There were significant seasonal differences in the frequency of carcass utilization by some scavenger species. For bears, carcass use significantly decreased from summer to autumn. In autumn, bears must accumulate energy for hibernation (e.g., Mclellan, 2011; Nelson et al., 1983) and they shift their primary food sources to nuts and acorns (Koike, 2010), which may be responsible for their changes in scavenging behavior between seasons. In contrast, the use of the carcasses by Japanese martens and mountain hawk‐eagles significantly increased from summer to autumn. Martens feed on insects principally in summer, but their intake of fruits increases as the availability of insects decreases in autumn (Koike & Masaki, 2019). Thus, martens may have increased their scavenging because of the decrease in their primary food sources. These results suggest that most omnivorous scavengers switch their feeding strategies by season in accordance with the availability of their primary food sources. Even the mountain hawk‐eagle, usually a predator, increased their scavenging of deer carcasses in autumn. A decrease in available reptiles, one of the primary food sources of the mountain hawk‐eagle, might also increase its scavenging in autumn. We did not place carcasses in winter, but carrion may be an important supplemental food resource in severe winters and influence the survival and reproduction of the scavengers (e.g., Houston, 1978, Angerbjörn, Arvidson, Norén, & Strömgren, 1991, Tannerfeldt, Angerbjorn, & ArvidSon, 1994). Thus, available carcasses in autumn might be an important food resource to enable scavengers to gain nutrition before winter and increase their probability of survival.

For some scavengers, including wild boar, raccoon dog, black kite, and jungle crow, the utilization of carcass did not change among seasons. The high utilization of carcasses by raccoon dogs suggest that they may be heavily dependent on nutrition from carrion in all seasons (e.g., Schlichting, Love, Webster, & Beasley, 2019; Selva et al., 2005), whereas the low utilization by wild boar, black kite, and jungle crow suggest they are not dependent on carcasses and are just opportunistic scavengers. The difficulties in detecting the carcasses for avian scavengers in mature forest may cause them to be more opportunistic and use carcasses as a random supplemental food source. On the other hand, scavengers that are heavily dependent on carcasses like raccoon dogs may be sensitive to variation in carcass availability. Additional studies are needed to evaluate the effects of carcass availability on their behavior, food habits, and reproductive parameters.

## CONFLICT OF INTEREST

The authors declare that they have no conflict of interest.

## AUTHOR CONTRIBUTIONS

AI and SK designed and coordinated the study. AI, MT, and TM performed the field work. AI, KY, and KT analyzed the data. AI, MA, and SK wrote the manuscript.

## Data Availability

The data used for this manuscript are available in the Illinois Data Bank, B2IDB https://doi.org/10.13012/B2IDB-9720784_V1.

## References

[ece35976-bib-0001] Allen, M. L. , Elbroch, L. M. , Wilmers, C. C. , & Wittmer, H. U. (2015). The comparative effects of large carnivores on the acquisition of carrion by scavengers. American Naturalist, 185(6), 822–833. 10.1086/681004 25996866

[ece35976-bib-0002] Allen, M. L. , Wilmers, C. C. , Elbroch, L. M. , Golla, J. M. , & Wittmer, H. U. (2016). The importance of motivation, weapons, and foul odors in driving encounter competition in carnivores. Ecology, 97(8), 1905–1912. 10.1002/ecy.1462 27859193

[ece35976-bib-0003] Angerbjörn, A. , Arvidson, B. , Norén, E. , & Strömgren, L. (1991). The effect of winter food on reproduction in the Arctic fox, Alopex lagopus: A field experiment. Journal of Animal Ecology, 60, 705–714. 10.2307/5307

[ece35976-bib-0004] Asahi, M. (1975). Contents of alimentary canals of weasels collected in Kinki district, with a consideration of their caloric nutrition. Zool Mag, 84, 190–195 (In Japanese with English abstract).

[ece35976-bib-0005] Beasley, J. C. , Olson, Z. H. , & DeVault, T. L. (2015). Ecological role of vertebrate scavengers In Benbow M. E., Tomberlin J. K., & Tarone A. M. (Eds.), Carrion ecology, evolution and their applications (pp. 107–127). Boca Raton, FL: CRC Press.

[ece35976-bib-0006] Blazquez, M. , Sanchez‐Zapata, J. A. , Botella, F. , Carrete, M. , & Eguía, S. (2009). Spatio‐temporal segregation of facultative avian scavengers at ungulate carcasses. Acta Oecol, 35(5), 645–650. 10.1016/j.actao.2009.06.002

[ece35976-bib-0007] Cortés‐Avizanda, A. , Jovani, R. , Carrete, M. , & Donázar, J. A. (2012). Resource unpredictability promotes species diversity and coexistence in an avian scavenger guild: A field experiment. Ecology, 93(12), 2570–2579. 10.1890/12-0221.1 23431588

[ece35976-bib-0008] Cortés‐Avizanda, A. , Selva, N. , Carrete, M. , & Donázar, J. A. (2009). Effects of carrion resources on herbivore spatial distribution are mediated by facultative scavengers. Basic and Applied Ecology, 10(3), 265–272. 10.1016/j.baae.2008.03.009

[ece35976-bib-0009] DeVault, T. L. , & Rhodes, O. E. (2002). Identification of vertebrate scavengers of small mammal carcasses in a forested landscape. Acta Theriologica, 47(2), 185–192.

[ece35976-bib-0010] DeVault, T. L. , Rhodes, O. E. Jr , & Shivik, J. A. (2003). Scavenging by vertebrates: And evolutionary on an important perspectives in terrestrial transfer energy pathway ecosystems. Oikos, 102, 225–234.

[ece35976-bib-0011] DeVault, T. L. , Seamans, T. W. , Linnell, K. E. , Sparks, D. W. , & Beasley, J. C. (2017). Scavenger removal of bird carcasses at simulated wind turbines: Does carcass type matter? Ecosphere, 8(11), e01994 10.1002/ecs2.1994

[ece35976-bib-0012] Fisher, R. A. (1934). Statistical methods for research workers (5th ed .). Edinburgh, UK: Oliver and Boyd.

[ece35976-bib-0013] Furuya, Y. , Kishida, R. , Swnoo, K. , Noguchi, K. , & Yamasaki, M. (1979). Seasonal changes of food habit of Weasels (Mustela sibirica) in Nishikuma Valley, Kochi Prefecture. Journal of the Mammalogical Society of Japan, 8(1), 1–11 (in Japanese with English abstract).

[ece35976-bib-0014] Gomo, G. , Mattisson, J. , Hagen, B. R. , Moa, P. F. , & Willebrand, T. (2017). Scavenging on a pulsed resource: Quality matters for corvids but density for mammals. BMC Ecology, 17(1), 22 10.1186/s12898-017-0132-1 28619108 PMC5472881

[ece35976-bib-0015] Hashimoto, Y. , & Takatsuki, S. (1997). Food habits of Japanese black bears: A review. Mammalian Science, 37, 1–19 (in Japanese with English abstract).

[ece35976-bib-0016] Hervē, M. (2019). RVAideMemoire: Testing and plotting procedures for biostatistics. Retrieved from https://cran.r-project.org/web/packages/RVAideMemoire/index.html

[ece35976-bib-0017] Higuchi, H. , Morioka, H. , & Yamagishi, S. (1996). The encyclogedia of animals in Japan, volume 3: Bird 1. Tokyo, Japan: Heibonsya (In Japanese).

[ece35976-bib-0018] Higuchi, H. , Morioka, H. , & Yamagishi, S. (1997). The encyclogedia of animals in Japan volume 4: Bird 2. Tokyo, Japan: Heibonsya (In Japanese).

[ece35976-bib-0019] Hothorn, T. , Bretz, F. , & Westfall, P. (2008). Simultaneous inference in general parametric models. Biometrical Journal: Journal of Mathematical Methods in Biosciences, 50(3), 346–363. 10.1002/bimj.200810425 18481363

[ece35976-bib-0020] Houston, D. C. (1978). Elk as winter‐spring food for carnivores in northern Yellowstone National Park. Journal of Applied Ecology, 15(3), 653–661. 10.2307/2402766

[ece35976-bib-0021] Hunter, J. S. , Durant, S. M. , & Caro, T. M. (2007). Patterns of scavenger arrival at cheetah kills in Serengeti National Park Tanzania. African Journal of Ecology, 45(3), 275–281. 10.1111/j.1365-2028.2006.00702.x

[ece35976-bib-0022] Inger, R. D. , Cox, T. C. , Per, E. , Norton, B. A. , & Gaston, K. J. (2016). Ecological role of vertebrate scavengers in urban ecosystems in the UK. Ecology and Evolution, 6(19), 7015–7023. 10.1002/ece3.2414 28725378 PMC5513233

[ece35976-bib-0023] Japan Meteorological Agency . (2016). Past weather data research. Tokyo, Japan: Author (In Japanese). Retrieved from http://www.data.jma.go.jp/gmd/risk/obsdl/index.php

[ece35976-bib-0024] Japan Meteorological Agency . (2017). Past weather data research. Tokyo, Japan: Author (In Japanese). Retrieved from http://www.data.jma.go.jp/gmd/risk/obsdl/index.php

[ece35976-bib-0025] Kaneko, Y. , Maruyama, N. , & Macdonald, D. W. (2006). Food habits and habitat selection of suburban badgers (*Meles meles*) in Japan. Journal of Zoology, 270(1), 78–89. 10.1111/j.1469-7998.2006.00063.x

[ece35976-bib-0026] Koike, S. (2010). Long‐term trends in food habits of Asiatic black bears in the Misaka Mountains on the Pacific coast of central Japan. Mamm Biol, 75(1), 17–28. 10.1016/j.mambio.2009.03.008

[ece35976-bib-0027] Koike, S. , & Masaki, T. (2019). Characteristics of fruits consumed by mammalian frugivores in Japanese temperate forest. Ecological Research, 34, 246–254. 10.1111/1440-1703.1057

[ece35976-bib-0028] Krofel, M. , Kos, I. , & Jerina, K. (2012). The noble cats and the big bad scavengers: Effects of dominant scavengers on solitary predators. Behavioral Ecology and Sociobiology, 66(9), 1297–1304. 10.1007/s00265-012-1384-6

[ece35976-bib-0029] Maki, H. , & Ohnishi, T. (2001). A photographic guide to the birds of Japan. Tokyo, Japan: Heibonsya (In Japanese).

[ece35976-bib-0030] Mammal Society of Japan . (2009). The guidelines for use and collection of animals in research (In Japanese). Retrieved from http://www.mammalogy.jp/guideline.html

[ece35976-bib-0031] Mateo‐Tomás, P. , & Olea, P. P. (2010). When hunting benefits raptors: A case study of game species and vultures. European Journal of Wildlife Research, 56(4), 519–528. 10.1007/s10344-009-0341-9

[ece35976-bib-0032] Mateo‐Tomás, P. , Olea, P. P. , Moleón, M. , Vicente, J. , Botella, F. , Selva, N. , … Sánchez‐Zapata, J. A. (2015). From regional to global patterns in vertebrate scavenger communities subsidized by big game hunting. Diversity and Distributions, 21(8), 913–924. 10.1111/ddi.12330

[ece35976-bib-0033] McCann, K. , Hastings, A. , & Huxel, G. R. (1998). Weak trophic interactions and the balance of nature. Nature, 395, 794–798. 10.1038/27427

[ece35976-bib-0034] Mclellan, B. N. (2011). Implications of a high‐energy and low‐protein diet on the body composition, fitness, and competitive abilities of black (*Ursus americanus*) and grizzly (*Ursus arctos*) bears. Canadian Journal of Zoology, 89(6), 546–558.

[ece35976-bib-0035] Ministry of the Environment . (2019). The protection and management of wild bird and mammal‐the capture number of Japanese sika deer and wild boar. Tokyo, Japan: Author (In Japanese). Retrieved from http://www.env.go.jp/nature/choju/docs/docs4/index.html

[ece35976-bib-0036] Mittermeier, R. A. , Myers, N. , Mittermeier, C. G. , & Robles, G. (1999). Hotspots: Earth's biologically richest and most endangered terrestrial ecoregions. Mexico: CEMEX, SA, Agrupación Sierra Madre, SC.

[ece35976-bib-0037] Moleon, M. , Sánche‐Zapata, J. A. , Margalida, A. , Carrete, M. , Owen‐Smith, N. , & Donázar, J. A. (2014). Humans and scavengers: The evolution of interactions and ecosystem services. BioScience, 64(5), 394–403. 10.1093/biosci/biu034

[ece35976-bib-0038] Moleón, M. , Sánchez‐Zapata, J. A. , Selva, N. , Donázar, J. A. , & Owen‐Smith, N. (2014). Inter‐specific interactions linking predation and scavenging in terrestrial vertebrate assemblages. Biological Reviews, 89(4), 1042–1054. 10.1111/brv.12097 24602047

[ece35976-bib-0039] Nelson, R. A. , Folk, G. E. , Pfeiffer, E. W. , Craighead, J. J. , Jonkel, C. J. , & Steiger, D. L. . (1983). Behavior, biochemistry, and hibernation in black, grizzly, and polar bears. Bears: their Biology and Management, 5, 284–290.

[ece35976-bib-0040] Neutel, A. M. , Heesterbeek, J. A. P. , & De Ruiter, P. C. (2002). Stability in real food webs: Weak links in long loops. Science, 296(5570), 1120–1123. 10.1126/science.1068326 12004131

[ece35976-bib-0041] Ohdachi, S. D. , Ishibashi, Y. , Iwasa, M. A. , & Saitoh, T. (2009). The wild mammals of Japan. Kyoto, Japan: SHOUKADOH Book Sellers and the Mammalogical Society of Japan.

[ece35976-bib-0042] Olson, Z. H. , Beasley, J. C. , & Rhodes, O. E. (2016). Carcass type affects local scavenger guilds more than habitat connectivity. PLoS ONE, 11(2), e0147798 10.1371/journal.pone.0147798 26886299 PMC4757541

[ece35976-bib-0043] Pardo‐Barquín, E. , Mateo‐Tomás, P. , & Olea, P. P. (2019). Habitat characteristics from local to landscape scales combine to shape vertebrate scavenging communities. Basic and Applied Ecology, 34, 126–139. 10.1016/j.baae.2018.08.005

[ece35976-bib-0044] Pereira, L. M. , Owen‐Smith, N. , & Moleón, M. (2014). Facultative predation and scavenging by mammalian carnivores: Seasonal, regional and intra‐guild comparisons. Mammal Review, 44(1), 44–55. 10.1111/mam.12005

[ece35976-bib-0045] R Core Team . (2016). R: A language and environment for statistical computing. Vienna, Austria: R Foundation for Statistical Computing.

[ece35976-bib-0046] Ray, R. R. , Seibold, H. , & Heurich, M. (2014). Invertebrates outcompete vertebrate facultative scavengers in simulated lynx kills in the Bavarian Forest National Park, Germany. Animal Biodiversity and Conservation, 37(1), 77–88.

[ece35976-bib-0047] Schlichting, P. E. , Love, C. N. , Webster, S. C. , & Beasley, J. C. (2019). Efficiency and composition of vertebrate scavengers at the land‐water interface in the Chernobyl Exclusion Zone. Food Webs, 18, e00107 10.1016/j.fooweb.2018.e00107

[ece35976-bib-0048] Sebastián‐González, E. , Barbosa, J. M. , Pérez‐García, J. M. , Morales‐Reyes, Z. , Botella, F. , Olea, P. P. , … Sánchez‐Zapata, J. A. (2019). Scavenging in the Anthropocene: Human impact drives vertebrate scavenger species richness at a global scale. Global Change Biology, 25(9), 3005–3017. 10.1111/gcb.14708 31127672

[ece35976-bib-0049] Sebastián‐González, E. , Moleón, M. , Gibert, J. P. , Botella, F. , Mateo‐Tomás, P. , Olea, P. P. , … Sánchez‐Zapata, J. (2016). Nested species‐rich networks of scavenging vertebrates support high levels of interspecific competition. Ecology, 97(1), 95–105. 10.1890/15-0212.1 27008779

[ece35976-bib-0050] Seki, Y. , Okuda, K. , & Koganezawa, M. (2014). Indirect effects of sika deer on japanese badgers. Mammal Study, 39(4), 201–208. 10.3106/041.039.0403

[ece35976-bib-0051] Selva, N. , & Fortuna, M. A. (2007). The nested structure of a scavenger community. Proceedings of the Royal Society of London. Series B: Biological Sciences, 274(1613), 1101–1108. 10.1098/rspb.2006.0232 17301021 PMC2124470

[ece35976-bib-0052] Selva, N. , Jędrzejewska, B. , Jędrzejewski, W. , & Wajrak, A. (2005). Factors affecting carcass use by a guild of scavengers in European temperate woodland. Canadian Journal of Zoology, 83(12), 1590–1601. 10.1139/z05-158

[ece35976-bib-0053] Sokal, R. R. , & Rohlf, F. J. (2012). Biometry: The principles and practice of statistics in biological research (4th ed .). New York, NY: W.H. Freeman and Company.

[ece35976-bib-0054] Sugiura, S. , & Hayashi, M. (2018). Functional compensation by insular scavengers: The relative contributions of vertebrates and invertebrates vary among islands. Ecography, 41(7), 1173–1183. 10.1111/ecog.03226

[ece35976-bib-0055] Sugiura, S. , Tanaka, R. , Taki, H. , & Kanzaki, N. (2013). Differential responses of scavenging arthropods and vertebrates to forest loss maintain ecosystem function in a heterogeneous landscape. Biological Conservation, 159, 206–213. 10.1016/j.biocon.2012.11.003

[ece35976-bib-0056] Takatsuki, S. , Suzuki, K. , & Suzuki, I. (1994). A mass‐mortality of Sika deer on Kinkazan Island, northern Japan. Ecological Research, 9(2), 215–223.

[ece35976-bib-0057] Tannerfeldt, M. , Angerbjorn, A. , & ArvidSon, B. (1994). The effect of summer feeding on juvenile arctic fox survival-A field experiment. Ecography, 17(1), 88–96.

[ece35976-bib-0058] Tochigi Prefecture . (2001). Birds of Tochigi Prefecture. Japan: Author (In Japanese).

[ece35976-bib-0059] Tochigi Prefecture . (2002). Mammals of Tochigi Prefecture. Japan: Author (In Japanese).

[ece35976-bib-0060] Tochigi Prefecture . (2018). Specified wildlife conservation and management plan – Deer. Japan: Author (In Japanese).

[ece35976-bib-0061] Turner, K. L. , Abernethy, E. F. , Conner, L. M. , Rhodes, O. E. , & Beasley, J. C. (2017). Abiotic and biotic factors modulate carrion fate and vertebrate scavenging communities. Ecology, 98(9), 2413–2424. 10.1002/ecy.1930 28628191

[ece35976-bib-0062] Wild Bird Society of Japan‐Tochigi (2015). A field guide to the birds of Tochigi. Tokyo: Mates Publishing (in Japanese).

[ece35976-bib-0063] Wilmers, C. C. , Crabtree, R. L. , Smith, D. W. , Murphy, K. M. , & Getz, W. M. (2003). Trophic facilitation by introduced trophic top predators: Grey wolf in Yellowstone National Park subsidies to scavengers. Journal of Animal Ecology, 72(6), 909–916.

[ece35976-bib-0064] Wilmers, C. C. , Stahler, D. R. , Crabtree, R. L. , Smith, D. W. , & Getz, W. M. (2003). Resource dispersion and consumer dominance: Scavenging at wolf‐ and hunter‐killed carcasses in Greater Yellowstone, USA. Ecology Letters, 6(11), 996–1003. 10.1046/j.1461-0248.2003.00522.x

[ece35976-bib-0065] Wilson, E. E. , & Wolkovich, E. M. (2011). Scavenging: How carnivores and carrion structure communities. Trends in Ecology & Evolution, 26(3), 129–135. 21295371 10.1016/j.tree.2010.12.011

[ece35976-bib-0066] Yamamoto, Y. (1994). Comparative analyses on food habits of Japanese marten, red fox, badger and raccoon dog in Mt. Nyugasa, Nagano Prefecture, Japan. Natural Environmental Science Research, 7, 45–52 (In Japanese).

[ece35976-bib-0067] Young, A. , Márquez‐Grant, N. , Stillman, R. , Smith, M. J. , & Korstjens, A. H. (2015). An Investigation of Red Fox (*Vulpes vulpes*) and Eurasian Badger (*Meles meles*) Scavenging, Scattering, and Removal of Deer Remains: Forensic Implications and Applications. Journal of Forensic Sciences, 60, S39–S55. 25065997 10.1111/1556-4029.12554

