# Supplementary material for: Vertebrate scavenger guild composition and utilization of carrion in an East Asian temperate forest
Source: Ecol Evol. 2020 Jan 21;10(3):1223–32. doi: 10.1002/ece3.5976 (PMC7029075; doi:10.1002/ece3.5976)
Supplement: Supplementary file 2 [file ECE3-10-1223-s002.docx]

**Appendix S1.** A video recording where an Asian black bear rubbed on a deer carcass repeatedly on 6 October 2017, a possible signal of dominance.
